# Treatment of a Canine Oral Papilloma With Topical Molecular Iodine: A Case Report With Implications for Antiviral Therapy in Humans

**DOI:** 10.7759/cureus.94049

**Published:** 2025-10-07

**Authors:** Mark Moskowitz

**Affiliations:** 1 Operative Dentistry, Lake Erie College of Osteopathic Medicine (LECOM) School of Dental Medicine, Bradenton, USA

**Keywords:** antiviral treatment, canine oral papilloma, molecular iodine, papillomavirus, veterinary case report

## Abstract

This case study presents the successful treatment and resolution of a canine oral papilloma using the topical application of molecular iodine. The observed efficacy of molecular iodine in this canine model raises translational relevance for human papillomavirus (HPV) and other viral infections, where current therapeutic options remain limited. Iodine has been shown to inactivate both bovine and HPV, the etiologic organisms responsible for papillomas in these species. A two-year-old dog exhibited nearly complete regression of a papillomatous lesion within three weeks of twice-daily application, and complete mucosal healing was observed clinically at four weeks. Molecular iodine is known to be virucidal against a wide range of viruses, including papillomavirus (PV). A canine oral papilloma was treated with a molecular iodine concentration of 300 ppm for one and a half minutes twice daily on a saturated paper towel. The papilloma steadily diminished in size until it was no longer detectable by clinical examination at four weeks. No recurrence was noted after one and a half years. This non-invasive, localized treatment may offer a simpler alternative to traditional surgical or systemic treatments for canine oral papillomas and underscores molecular iodine as a potential adjunctive therapy with implications for both animal and human health.

## Introduction

Existing literature describes invasive, lengthy, and expensive treatments for canine papillomas. Such treatment options include surgery [1], intramuscular injections of Anthiomaline-Lithium antimony thiomalate [2], cryotherapy, surgery and chemotherapeutic management with an electric thermo-cauterizer [3], azithromycin antibiotic therapy [4], a homeopathic combination of Sulfur 30, Thuja 30, Psorinum 30, and Graphites 30 given orally [5], and an inactivated virus administered via subcutaneous injection in combination with oral administration of *Thuja occidentalis *[6]. All of these treatments have their drawbacks, including costs, multiple trips to the medical facility, postoperative wound management, potential systemic issues, and the possible need for sedation. 

This case report highlights a novel treatment for a single canine oral papilloma with a 90-second application of a 300 ppm topical molecular iodine solution, twice daily. The site of the lesion exhibited no recurrence over one and a half years. The reaction of the iodine disinfectant mainly involves the structural proteins of the capsid, the tyrosine or histidine residues in the proteins [7]. Much of the current scientific literature shows studies utilizing povidone iodine (PVP-I) as a source of molecular iodine for disinfection and treatments. PVP-I, better known by the trade name Betadine, is a complex of polyvinylpyrrolidone (povidone) and triiodide ions that is widely used as an antiseptic for skin, mucous membranes, and wounds [8]. The molecular iodine treatment of a canine papilloma presented in this case report highlights a novel, simple, inexpensive, non-invasive, and rapid treatment, which did not result in a postoperative wound or systemic effects. This treatment may have potential benefits to animals and humans for other pathologies of viral etiology. 

## Case presentation

Molecular iodine was chosen for the treatment of a canine oral papilloma. Molecular iodine is frequently used to reduce bacterial levels in both humans and animals prior to surgery [7]. Papillomas are caused by the canine papillomavirus (CPV), which is a non-enveloped virus [9,10]. Antiseptic solutions containing molecular iodine, such as Betadine and other PVP-I brands, have demonstrated a 99.99% efficacy against both enveloped and non-enveloped viruses [11].

The antiviral efficacy of these PVP-I solutions is directly related to the amount of free molecular iodine present [12]. The highest level of free molecular iodine achievable in PVP-I solutions has been documented to be 25 ppm. Ironically, this occurs only in the diluted solutions, and the amount of free iodine present in the more concentrated solutions is much lower [13]. It has been shown that even higher concentrations of free molecular iodine are more efficacious as disinfectants. Concentrations of 20-175 ppm destroyed *Bacillus subtilis* spores as well as *Staphylococcus aureus* even faster than PVP-I, with its lower concentration of free molecular iodine [14]. Since the virucidal efficacy of iodine solutions is directly related to the concentration of free molecular iodine, there is potentially great clinical value for topical applications of higher concentrations [13]. A solution of molecular iodine at a concentration of 300 ppm was chosen for the treatment of this canine papilloma because it was significantly higher in free molecular iodine concentration than PVP-I and readily available. The molecular iodine solution used in this report is ioVet Oral® (ioTech International, Florida, USA) and has a maximum concentration of molecular iodine of 300 ppm when undiluted.

**Figure 1:**
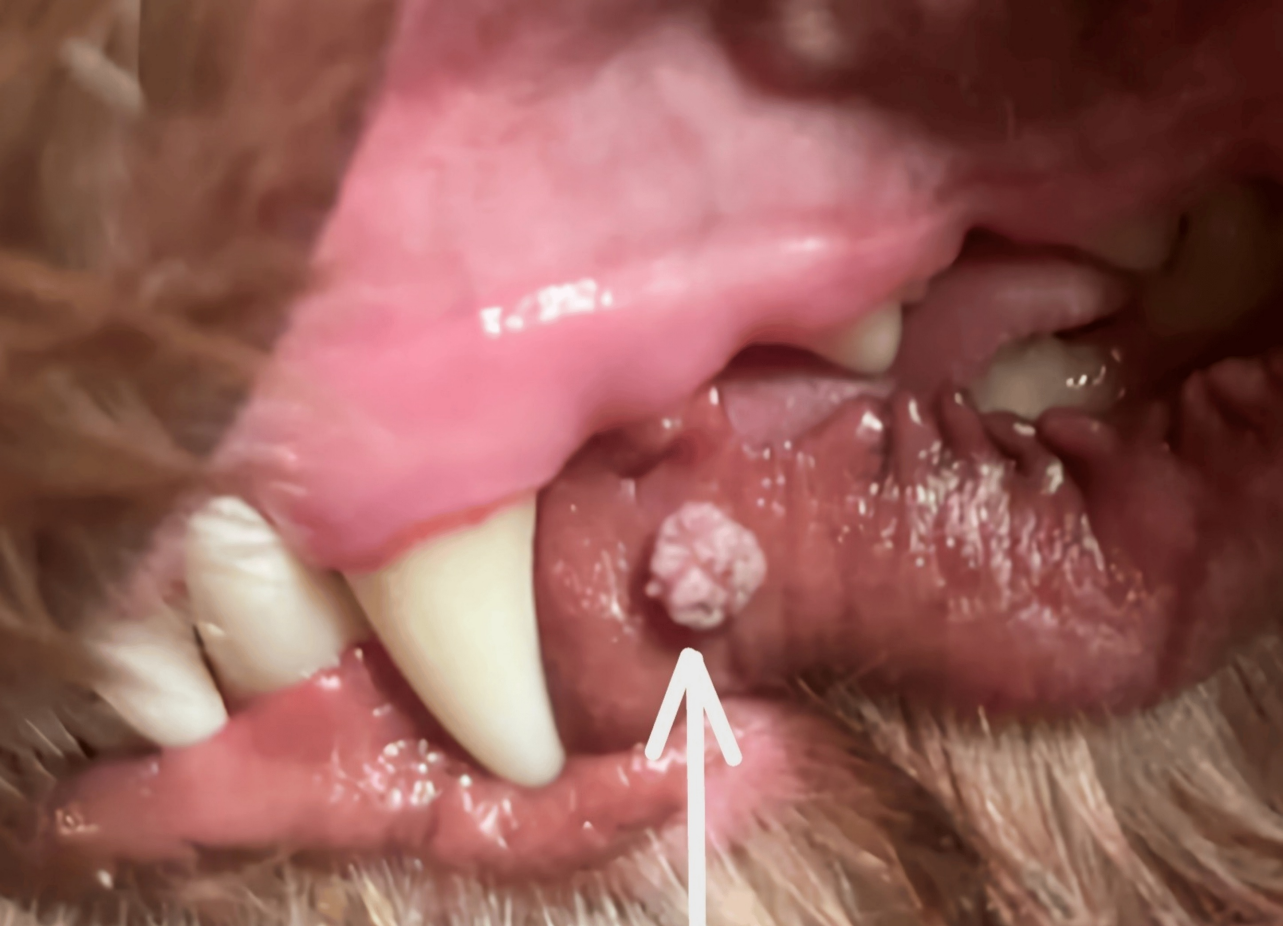
Canine oral papilloma at the time of discovery on the outer mucosa of the left lower lip (arrow). The lesion is endophytic with a verrucous or cauliflower-like surface and measures 7 mm in diameter.

The papilloma was found in a two-year-old, healthy, fifty-five-pound, male, Australian Shepherd-Poodle mixed breed. The lesion was not present very long before it was noticed, as the owner was in the habit of brushing the dog’s teeth twice daily. The dog resided in a private residence as a family pet and interacted once daily with neighborhood dogs in a local dog park with a shared drinking fountain and shared tennis balls to play with. The dog was not on any immunosuppressive medications and was considered quite healthy. The papilloma was present on the external mucosal surface of the lower left lip and measured approximately 7 mm in diameter. Examination of the mouth revealed only one lesion present. The lesion had a verrucous or cauliflower-like surface texture and was exophytic, as shown in Figure 1.

Papillomaviruses (PVs) have an affinity for epithelial cells and proliferate in these cells. As they multiply in epithelial cells, they result in an increase in the epithelial layer, which results in the folding of this widened layer of the epithelium and the morphologic appearance of the cauliflower-like papilloma. When viruses multiply in the epithelial cells, cells tend to enlarge, and the nuclei appear shrunken due to masking by cytoplasm. This appearance is referred to as koilocytes and resembles a halo around the nucleus. Nuclei may become enlarged and irregular in shape. PV-infected cells often exhibit keratohyalin granules that are clumped together [15].

The lesion treated in this case report was not examined histologically, since it resolved completely with the treatment presented here. Papillomas occur most commonly on the oral mucosa or at the junction of the mucosa and the dermis. Papillomas have a pathognomonic presentation in their exophytic and cauliflower-like morphology, and as a result, can be diagnosed clinically [9]. Since the papilloma is so obvious in its appearance, diagnosis is accomplished without laboratory testing in young dogs [16].

**Figure 2:**
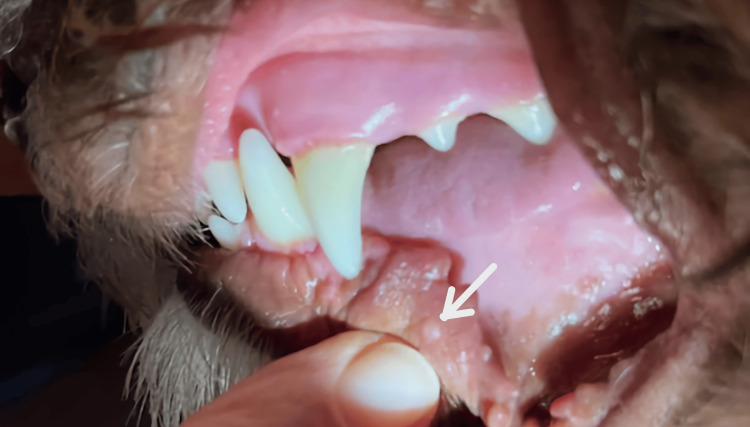
A small remnant of the lesion is shown after three weeks of molecular iodine treatment (arrow).

Treatment of the papilloma was accomplished by saturating a small piece of paper towel with molecular iodine 300 ppm (ioVet Oral® undiluted by ioTech International) and holding it on the lesion twice daily for 90 seconds by the dog's owner. The treatment was suggested by a dentist and not reviewed with a veterinarian. The molecular iodine was applied to the lesion at its full undiluted strength of 300 ppm for maximum antiviral effectiveness. This is a patented form of molecular iodine, the principal biocidal form of iodine, in which the free molecular iodine concentration is approximately 100 times greater than that of PVP-I. It has been shown that molecular iodine is not cytotoxic at concentrations much higher than those found in PVP-I [13]. The adjacent mucosal tissues did not experience any adverse effect clinically, and no systemic effects were noted. The lesion shrank to half of its original size after one week of treatment and was nearly gone by the end of three weeks, as shown in Figure 2.

**Figure 3:**
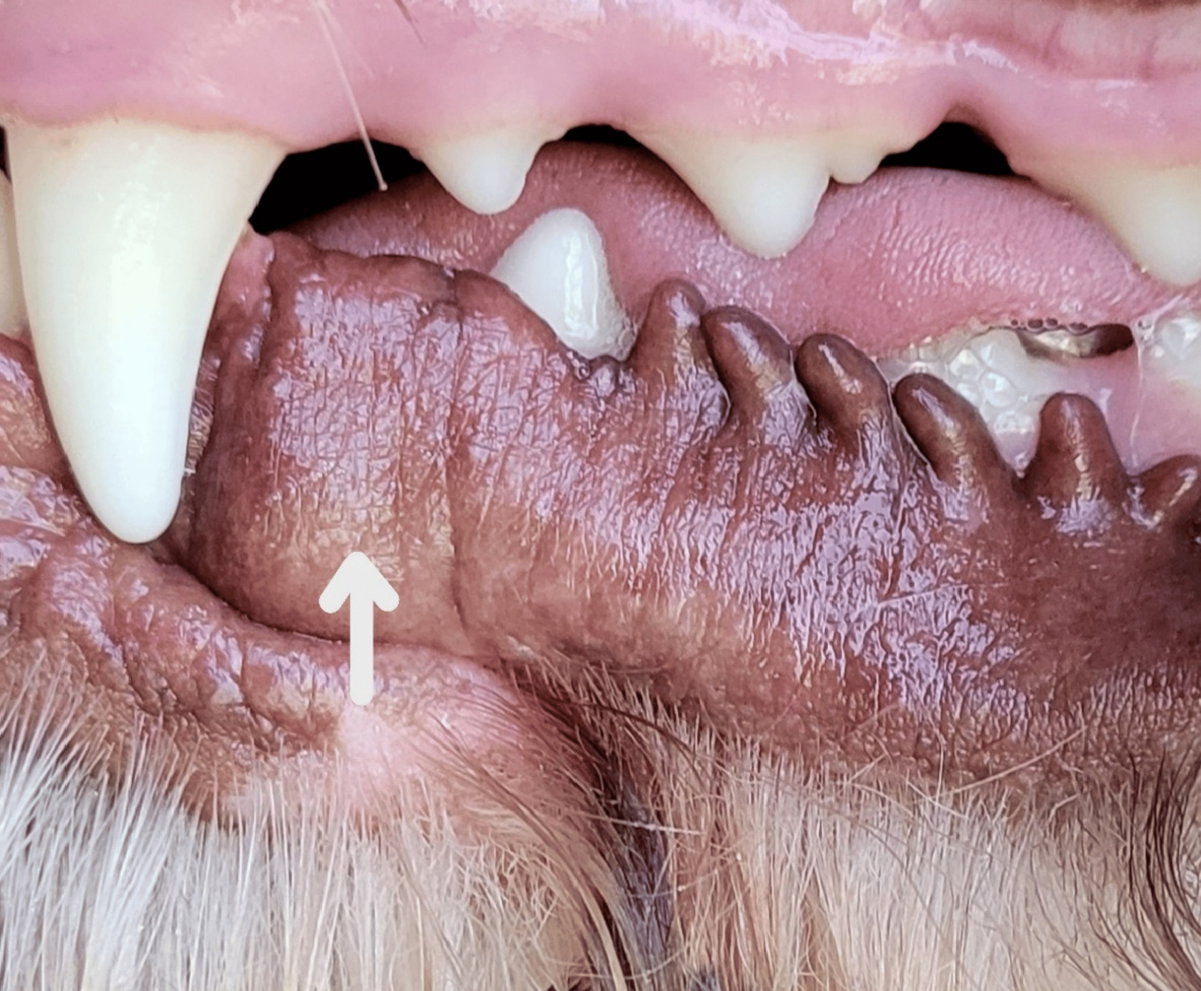
The lesion site is shown four weeks after treatment with molecular iodine 300 parts per million (arrow). Tissue appears clinically healthy with no sign of recurrence.

 At four weeks, the mucosa appeared clinically healthy with no sign of the lesion, as shown in Figure 3.

**Figure 4:**
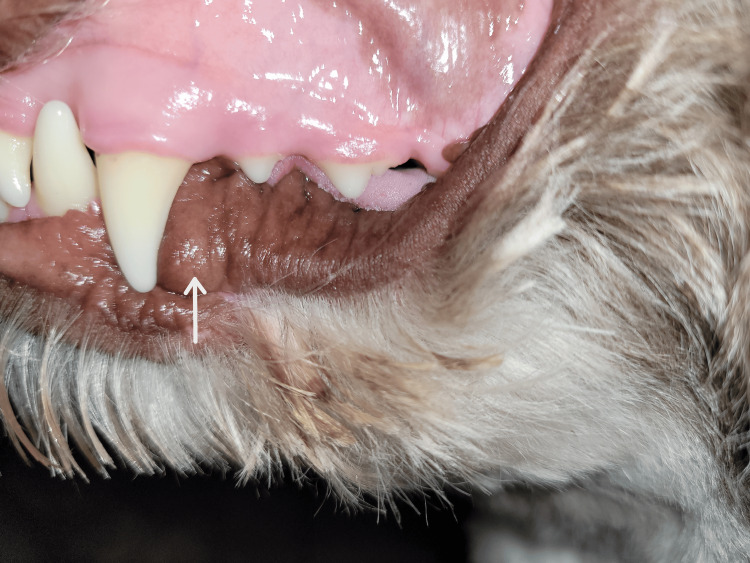
The lesion site is shown one and a half years following treatment with molecular iodine (300 ppm), with no clinical sign of recurrence (arrow).

No recurrence was detected clinically after one and a half years, as seen in Figure 4. Informed consent was obtained from the animal’s owner for publication of this case report.

## Discussion

In this case report, a canine oral papilloma is treated successfully with molecular iodine. Papillomas in dogs are caused by the CPV [15]. The structure of the PV presents double-stranded DNA. The genome is circular, and there are approximately 8000 base pairs. The virus is lacking an envelope and has an icosahedral capsid [16]. At the time of this case report, the PV episteme lists a total of 33 CPVs [17]. The CPV that causes most oral and some cutaneous lesions is CPV1 [9]. It is difficult to assess how often PV infection occurs, since many cases are likely not recorded. However, one study showed that dogs in South Africa had canine oral PV (COPV) antibodies at 29%, compared to Switzerland, which displayed a seroprevalence of only 10.5% [18]. Another study revealed that subclinical CPV shedding is likely [19]. Therefore, it is prudent to keep animals that have never been infected away from those with a history of infection [15]. CPV is possibly even transmitted from what seems like the uninfected skin and oral mucosa of the infected dog [10].

Pathogenic potential

The papilloma resulting from infection with CPV1 is a benign lesion. Early detection and treatment until complete regression is beneficial to reduce risks of squamous cell carcinoma; however, if not detected early, it serves as a risk factor for the development of squamous cell carcinoma, as the risk for malignant transformation may exist [20]. Most commonly, this pathology regresses within five months; however, sometimes intervention is necessary, and more invasive procedures may be required [21]. Squamous cell carcinomas have shown the presence of CPV in approximately 2.3% of lesions in a sample of 212 cases, and there is a potential possibility of malignant transformation in dogs [22]. The rate of this transformation in one study was found to be 13 dogs out of a sample of 365, or 3.6% [20].

Speed of resolution

Treatments to speed up the resolution of papillomas have been attempted with varying degrees of success. Munday noted that in a double-blind study involving the treatment of canine papillomas in 17 dogs with systemic azithromycin, lesions regressed after 50 days for the 10 dogs that were treated, and regression only occurred in one of the non-treated dogs [4,15].

In a study of forty canines, it was noted that regression times varied. In 11 of the dogs, the papillomas took three months to regress, and in five of the dogs, the regression took more than three months and even up to a year [9,15].

In the present case report, utilizing molecular iodine at a concentration of 300 ppm (ioVetOral® from ioTech International), after two weeks, the lesion regressed to less than half its original size, and after three weeks, it was nearly gone. This is a more rapid resolution than the 12 months described for untreated papillomas and the more lengthy and variable 50 days to a year for those treated with systemic azithromycin [4].

Invasiveness of treatment

Many pet owners choose to have papillomatous lesions removed because they are unsightly. However, this can be dangerous for older dogs with decreased liver function and failing kidneys. Excisional biopsy for purely esthetic purposes is not recommended for older animals because of the elevated risk of systemic anesthetic agents [1]. Surgical approaches often require anesthesia and carry risks of infection and prolonged healing. Additional steps must often be taken to prevent the animal from disturbing the surgical wound. The administration of inactivated virus via subcutaneous injection in combination with oral administration of *Thuja occidentalis* was found to be effective in one canine subject over a four-week course of treatments [6]. However, this involved extensive preparation and administration of four injections over four veterinarian visits. Systemic antibiotics such as azithromycin are sometimes associated with unwanted side effects such as nausea. Topical application of molecular iodine is a non-invasive treatment that can easily be performed by pet owners while avoiding the potential complications that may come with more invasive or systemic treatments. The concentration of molecular iodine used in this case report is readily available to consumers. 

Mechanism of action

It has not yet been determined which antigens stimulate the immune system response to the papilloma. However, CPV infection of the oral mucosa of dogs is a translational example of PV infection resulting in papillomatous lesions. This permits us to better comprehend the immune responses to the infection. Early T cell reaction to the E2 protein and an increase in the number of lymphocytes are apparent and indicative of the immune response. A reduction in the T cell response happens quickly when the papilloma regresses [23]. CD4+ and CD8+ lymphocytes are detectable in higher numbers just prior to resolution [24]. Molecular iodine may help to activate the phagocytic cells, which may lead to an increase in cytokines and hence, the arrival of even greater numbers of immune cells [25].

After receiving xenografts, mice that were given supplemental molecular iodine had higher numbers of white blood cells and larger numbers of lymphocytes in the tumors while demonstrating CD8+ activation responses against the tumors [26].

Molecular iodine is known to be extremely bactericidal, virucidal, and fungicidal. In one study, a 100 ppm molecular iodine rinse demonstrated nearly complete destruction of SARS-CoV-2 after only 30 seconds, even in the presence of human saliva [27]. This suggests that molecular iodine possesses a potential for broad-spectrum antiviral applications.

Even at low concentrations of molecular iodine, PVP-I has destroyed bovine PV [28]. The free molecular iodine concentrations were found to be the highest in the more diluted solution of PVP-I (0.17 ppm), which corresponded to a 0.1% solution. Molecular iodine has been shown to destroy poliovirus type 1 and adenovirus type 3, and influenza A virus was no longer viable after only 15 seconds of exposure time. These data show a direct correlation between the concentration of free molecular iodine and virucidal potency [12]. Therefore, the clinical advantages of higher levels of free molecular iodine are greater [13].

**Figure 5:**
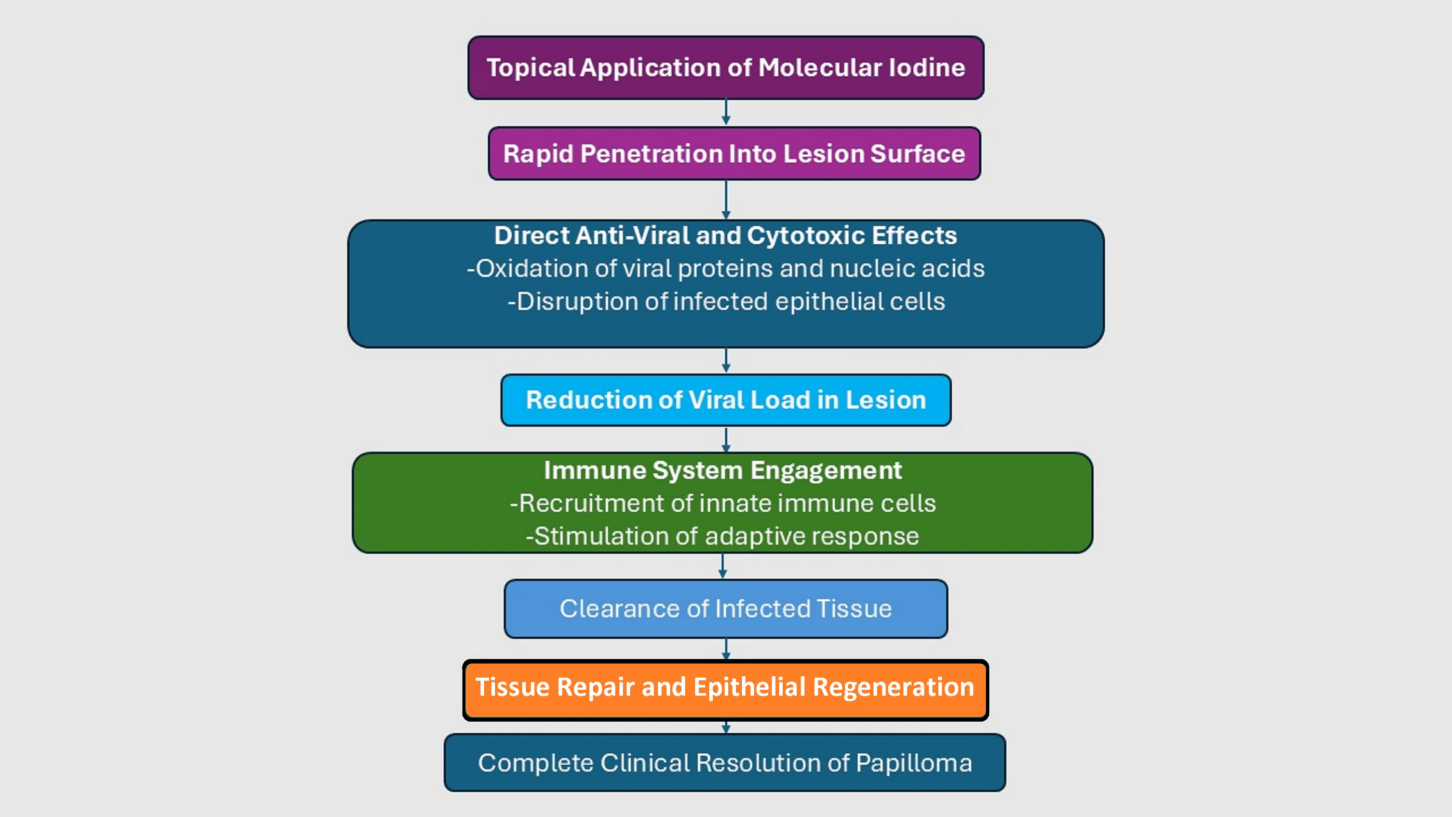
Proposed mechanism of action of molecular iodine in oral papilloma resolution. Topical molecular iodine penetrates the lesion surface, producing direct antiviral and cytotoxic effects through oxidation of viral proteins and nucleic acids and disruption of infected epithelial cells [7,11]. This reduces viral load and facilitates immune system engagement, including recruitment of innate immune cells and stimulation of adaptive immunity. The combined effects result in clearance of infected tissue, followed by epithelial repair and complete clinical resolution of the papilloma [25,26]. Original schematic created by the author. Mechanistic steps informed by previously published data [7,11,25,26].

PVP-I has been shown to destroy many types of viruses. The way in which it accomplishes this resembles the way it destroys many kinds of bacteria. Free molecular iodine is a potent oxidizer that affects essential parts of the organisms, such as membranes and amino acids. Even viral enzymes like neuraminidase are affected, preventing the release of viruses outside of the infected cell, thereby inhibiting the continued invasion of other healthy cells. In addition, PVP-I may also block hemagglutinin, preventing the binding of viruses to healthy cells [11]. The proposed mechanism by which molecular iodine promotes papilloma resolution is illustrated in Figure 5.

Another study showed that tumors exposed to molecular iodine became less invasive. A higher number of tumor cell deaths and increased immune responses were detected [28]. In a solution of .1% of PVP-I, the concentration of free molecular iodine can reach its highest level, which is approximately 25 ppm [29]. The 300 ppm concentration of molecular iodine used in this case report is significantly higher than that found in PVP-I, suggesting a stronger antiviral effect. These effects could support the rapid resolution of the canine oral papilloma observed in this case. Additional double-blind controlled studies with a larger sample size should be done to better assess the efficacy of molecular iodine on the treatment of canine papillomas and its potential effects on epithelial cells in general.

Potential benefit to humans

The canine papilloma is an important model of mucosal human PV (HPV) infections. PVs occasionally cause severe, non-regressing, or recurrent infections in their human and animal hosts. The lymphocyte infiltrate in the dog resembled that in HPV lesions, indicating that COPV is an appropriate model for HPV immunity [24]. In humans, a majority of benign verrucous papillary lesions have a viral etiology [30]. HPV-related lesions of the oral cavity include squamous papilloma, condyloma acuminatum, verruca vulgaris, and multifocal epithelial hyperplasia [31]. PVP-I has shown promise in the treatment of other viral conditions. Approximately 90% inactivation of PV was demonstrated with exposure to 0.1% PVP-I, and 99.9% inactivation was seen at 0.3%. Clinical trials may be warranted to determine whether PVP-I would reduce the rate of sexual transmission of the HPV strains associated with cervical cancer [32]. PVP-I-based oral and nasal preparations showed favorable results in terms of reducing SARS-CoV-2 viral loads both in vivo and in vitro [33]. Conjunctival irrigation with 2.5% PVP-I is effective in the treatment of adenoviral conjunctivitis in infants [34]. Cryotherapy, accompanied by the application of PVP-I, is effective in the prophylaxis and eradication of persistent infection with highly oncogenic HPV [35]. PVP-I paint was helpful in treating symptoms of herpes zoster and herpes simplex [36]. PVP-I was found to rapidly inactivate a range of viruses, such as adeno-, mumps, rota-, polio- (types one and three), coxsackie-, rhino-, herpes simplex, rubella, measles, influenza, and human immunodeficiency viruses. Chlorhexidine gluconate, alkyldiaminoethyl-glycine hydrochloride, benzalkonium chloride, and benzethonium chloride were ineffective against herpes-, adeno-, polio-, and rhinoviruses [37]. Higher concentrations of free molecular iodine than those found in PVP-I may be even more efficacious against these and other viral pathogens.

## Conclusions

Molecular iodine (300 ppm) may be a non-invasive, cost-effective, simple, and inexpensive treatment option for canine oral papillomas. This case report utilized a molecular iodine concentration that was significantly higher than the concentration of free molecular iodine found in PVP-I. This concentration of molecular iodine was effective in treating a papilloma caused by CPV. Limitations of this case report include a small sample size of one papilloma, no opportunity for histological examination, and no polymerase chain reaction confirmation of CPV. Further research should investigate the mechanism of action for molecular iodine on canine oral papilloma, as well as investigate the potential benefit of molecular iodine for the treatment of similar lesions in both animals and humans. Since the virucidal efficacy of iodine solutions increases with the concentration of free molecular iodine, solutions with higher concentrations of free molecular iodine than those found in PVP-I may be helpful in treating or reducing the spread of other diseases of viral etiology in both animals and humans. Examples of such diseases and viral pathogens include viral conjunctivitis, otitis media, pharyngitis, sinusitis, chickenpox, shingles, herpes, HIV, influenza, norovirus, HPV, SARS-CoV-2, and the common cold.
